# Automated Classification of Neuromuscular Diseases Using Thigh Muscle MRI With Model Interpretations

**DOI:** 10.1002/jcsm.70102

**Published:** 2025-10-11

**Authors:** Lotte Huysmans, Louise Iterbeke, Bram De Wel, Matthias Opsomer, Kristl G. Claeys, Frederik Maes

**Affiliations:** ^1^ Medical Imaging Research Centre University Hospitals Leuven Leuven Belgium; ^2^ Department ESAT – PSI KU Leuven Leuven Belgium; ^3^ Laboratory for Muscle Diseases and Neuropathies, Department of Neurosciences KU Leuven, and Leuven Brain Institute (LBI) Leuven Belgium; ^4^ Department of Neurology University Hospitals Leuven Leuven Belgium

**Keywords:** disease classification, neuromuscular diseases, quantitative MRI, SHAP explanations

## Abstract

**Background:**

Neuromuscular diseases (NMDs) are diagnosed using a combination of clinical evaluation, electromyography, nerve conduction studies, blood tests, muscle biopsy, and genetic testing. In addition, muscle magnetic resonance imaging (MRI) is used to visualise affected areas and allows the identification of fatty replacement of muscle tissue, muscle atrophy and oedema. The distinct muscle involvement patterns can be used to help in the diagnosis of NMDs. Our aim was to develop an automatic approach with interpretations that explain the model's decision to classify NMDs based on symptomatic MRI scans of the upper leg.

**Methods:**

We used 109 Dixon muscle MRI scans of the upper legs of four different NMDs: limb–girdle muscular dystrophy type R12 (LGMDR12), Becker muscular dystrophy (BMD), myotonic dystrophy type 1 (DM1), Charcot–Marie–Tooth neuropathy type 1A (CMT1A) and healthy controls (HC). A U‐Net was trained to segment all 18 muscles in the upper leg from which the fat fractions are calculated and used as input to a random forest classification model. SHapley Additive exPlanations (SHAP) are used to get an understanding of the reasoning of the model and are compared with muscle involvement patterns previously described in the medical literature.

**Results:**

The baseline models demonstrate strong performance in distinguishing between different classes, as evidenced by an overall accuracy of 89% and high area under the receiver operating characteristic curve (AUC) values for every class: 0.972, 0.983, 0.960, 0.990 and 0.997 for respectively LGMDR12, BMD, DM1, CMT1A and HC. In addition, we demonstrated that no significant difference could be observed with models trained on features calculated from ground truth segmentations, features calculated from a limited field of view or Mercuri score features. SHAP explanations help understand the decision of the models and can be linked to muscle patterns described in the medical literature.

**Conclusion:**

A fully automated method was developed that is effective in distinguishing between four NMDs and healthy controls.

## 1 Introduction

The diagnosis of NMDs involves a combination of clinical evaluation, electromyography, nerve conduction studies, blood tests, muscle biopsy and/or genetic testing. In addition to these, imaging techniques such as muscle magnetic resonance imaging (MRI) are often used to visualise affected areas. MRI allows the identification of fatty replacement of muscle tissue, muscle atrophy and oedema. It can also be used for the diagnosis of diseases that have distinctive patterns of muscle involvement on MRI, such as in limb–girdle muscular dystrophy type R12 (LGMDR12), Becker muscular dystrophy (BMD), Charcot–Marie–Tooth disease type 1A (CMT1A), and myotonic dystrophy type 1 (DM1). The involvement of specific thigh muscles as reported in the literature is summarised in Table 1 for these four diseases [1, 2, 3, 4, 5, 6, 7].

**TABLE 1:** Different NMDs included in this study, thigh muscle involvement as reported in the literature for each NMD, number of cases available in this study and number of cases included in the training set for muscle segmentation and NMD classification. LGMDR12: Limb‐Girdle Muscular Dystrophy type R12; BMD: Becker Muscular Dystrophy; DM1: Myotonic Dystrophy type 1; CMT1A: Charcot–Marie–Tooth disease type 1A; HC: healthy controls.

NMD	Affected	Spared	# cases	# used for training
LGMDR12	Adductor magnus, semitendinosus, semimembranosus	Flutei, gracilis, sartorius	24	15
BMD	Adductor magnus, gluteus medius, gluteus maximus, biceps femoris caput longus	Adductor longus, gracilis, sartorius	21	14
DM1	Vastus intermedius, vastus medialis	Rectus femoris, gracilis	32	19
CMT1A	Biceps femoris, gracilis, semitendinosus, semimembranosus	Adductor longus, adductor magnus, vastus intermedius, vastus medialis	30	14
HC	None	All	49	47
Total			156	109

Díaz‐Manera [2] analysed the muscle MRI pattern of various NMDs by manually grading every muscle on T1‐weighted axial MR images using the Mercuri grading score modified by Fischer et al. [8, 9]. Verdú‐Díaz et al. [10] used these scores for 70 pelvic, thighs and legs muscles of 976 patients to train a random forest classifier for discriminating between 10 different NMDs, reporting an accuracy of 95.7%. The drawback of this semiquantitative approach to discriminate different NMDs based on their MRI pattern is that manual scoring of all muscles considered is required, which is time‐consuming and subjective. Cai et al. [11], Fabry et al. [12] and Yang et al. [13] applied deep learning using different convolutional neural network (CNN) architectures to classify the MRI scans directly according to disease type. While their approaches are fully automated, these works only differentiated between not more than two NMDs and the CNN largely acts as a black box whose outcome is difficult to interpret.

In this work, we introduce a fully automatic and interpretable approach to discriminate between different NMDs based on the muscle MRI pattern in the thighs. A CNN is trained to automatically segment the 18 muscles present in the proximal leg in the MRI scans of normal appearing and symptomatic NMD cases, which allows us to calculate the mean percentage of fat fraction (FF%) over the full 3D volume of each muscle. These FF% features are subsequently used as input to train a random forest classifier to identify each symptomatic case as a particular NMD. SHAP values [14] are used to explain the output of the classifier, allowing the physician to interpret the outcome in terms of the degree of involvement of specific muscles.

## 2 Materials and Methods

### 2.1 Patients and MRI Datasets

Four datasets of MRI scans of the proximal legs were used in this study, acquired in different clinical studies involving different NMD patients and healthy controls (HC). The first dataset consisted of 24 patients with LGMDR12 and 27 HC [15], and the second included 21 patients with BMD and another 22 HC [16]. These two natural history studies were initiated first and focused on disorders known to predominantly affect proximal muscles. The third dataset comprised 30 CMT1A patients, and the fourth included 32 DM1 patients; these natural history studies were initiated later and involve disorders that typically affect distal, and to a lesser extent, proximal muscles. Written informed consent was obtained from all participants, and the studies were approved by the Ethics Committee Research UZ/KU Leuven and performed according to the relevant guidelines and regulations.

**FIGURE 1:**
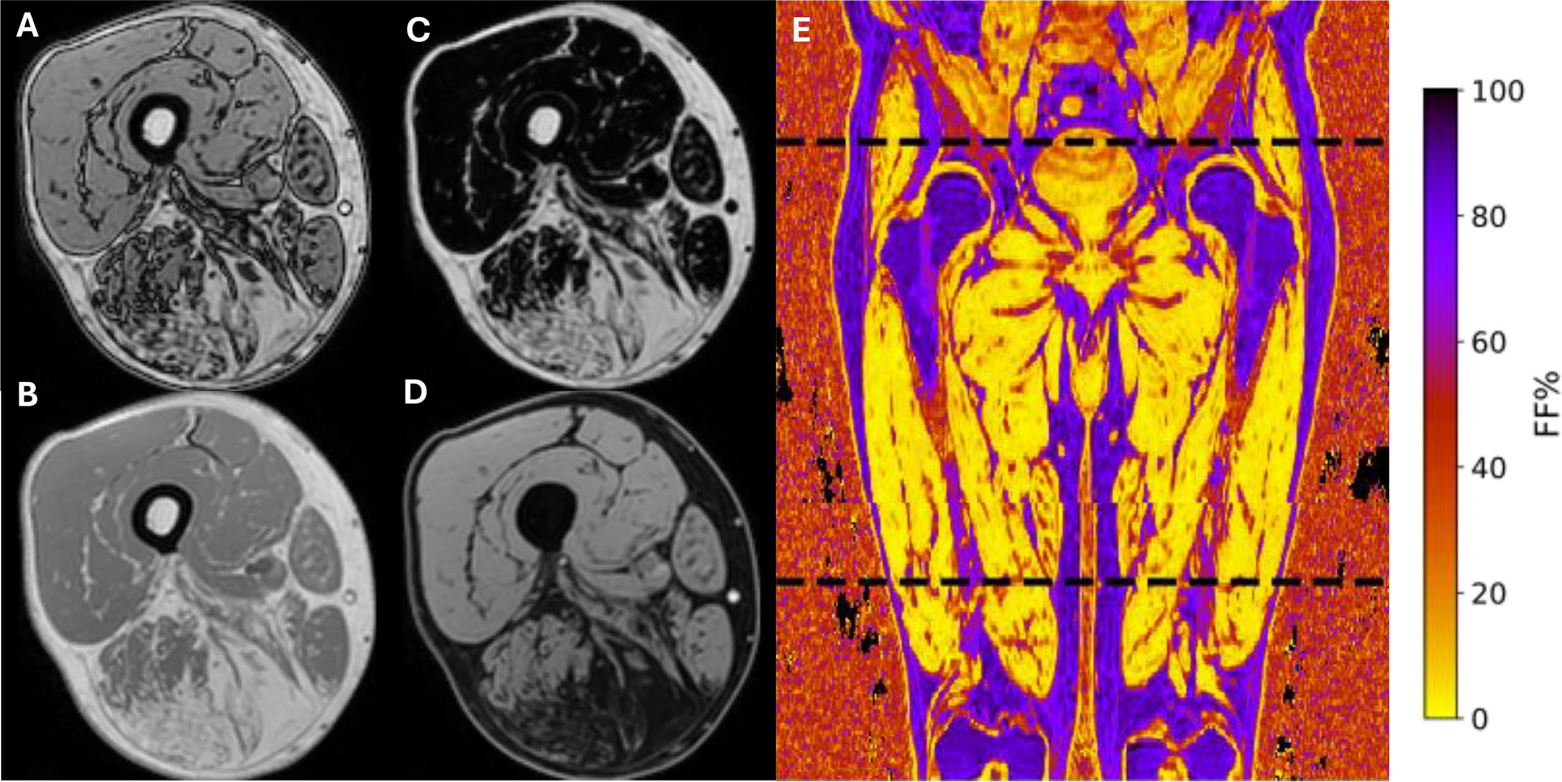
Dixon MRI images. Axial slice of the out‐of‐phase (A), in‐phase (B), fat‐only (C) and water‐only (D) images of a LGMDR12 patient and coronal slice (E) showing the PDFF image with the full field of view. The limited field of view is indicated by the black dotted lines.

MRI scans of BMD, LGMDR12 and HC cases were acquired with a 1.5‐T MRI scanner (Philips Ingenia, Philips Medical Systems) using a 6‐point Dixon 3D imaging sequence [17]. Postprocessing included a correction for T1 weighting as described by Liu et al. [18]. MRI scans of the DM1 and CMT1A cases were acquired with a 3‐T MRI scanner (Philips Ingenia, Philips Medical Systems). No correction for T1 weighting was necessary. All patients were scanned in a supine, feet‐first position. The detailed protocol parameters are summarised in Table 2. Three axial image stacks of the thighs [overlapping by 20 (1.5 T) or 25 (3 T) slices] were acquired to capture all thigh muscles from origin to insertion, and the stacks were stitched together to form one image volume. The 6‐point Dixon protocol results in six echo images from which in‐phase (IP), out‐of‐phase (OP), fat‐only (F) and water‐only (W) images were reconstructed by the Philips scanner using a seven‐peak spectral fat model [19]. The proton density fat fraction image (PDFF) was then calculated by dividing the F image by the sum of the F and W images, providing a quantitative measure for the amount of fat present in each voxel, as illustrated in Figure 1. Conventional T1‐weighted images were also available for each patient. Detailed protocol parameters are provided in Table 2.

**TABLE 2:** Dixon protocol and T1 protocol.

	6‐point Dixon	T1
BMD, LGMDR12, HC	TR/TE/δTE = 9.2/1.36/1.3 ms, 12° flip angle, 140 slices, 2 mm slice thickness, FOV = 384 × 384, voxel size = 1.2 × 1.2	T1 turbo spin echo, TR/TE = 412/4 ms, 30 slices, 8 mm slice thickness, 1 mm interslice gap, FOV = 300 × 455 mm, voxel size = 1.4 × 1.4
CMT1A, DM1	TR/TE/δTE = 10/1.50/1.03 ms, 3° flip angle, 130 slices, 2 mm slice thickness, FOV = 384 × 384, voxel size = 1.2 × 1.2	T1 turbo spin echo, TR/TE = 546/20 ms, 30 slices, 8 mm slice thickness, 1 mm interslice gap, FOV = 301 × 455, voxel size = 0.8 × 0.8

The acquisition of three Dixon MRI stacks to capture a sufficiently large field of view (FOV) that includes all 18 thigh muscles from origin to insertion may be too time‐consuming in the routine clinical setting. Each Dixon stack takes approximately 6 min to acquire, resulting in a total scan time of around 18 min. Hence, the feasibility of discriminating different NMDs using a single MRI stack providing only partial muscle information was investigated. To this end, a stack of slices was extracted from the original images, with its first slice positioned at the proximal end of the sartorius and extending 320 mm towards the distal end of the upper leg. This position was chosen because most of the muscles are (at least partially) visible within this limited FOV.

### 2.2 3D Muscle Segmentation

**FIGURE 2:**
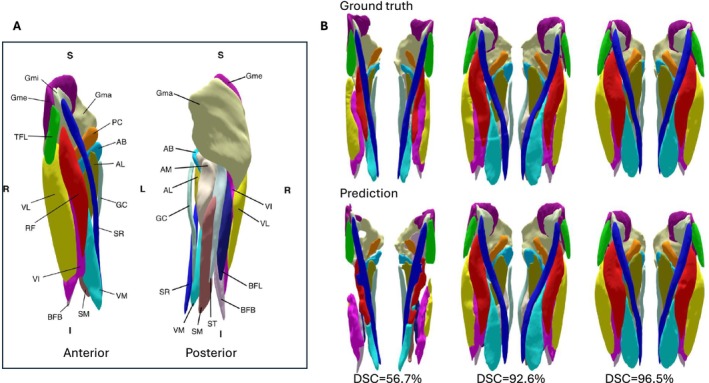
Illustration of 3D muscle segmentation quality. Panel A: Anterior and posterior views of the 18 proximal leg muscles. Adductors: adductor magnus (AM), adductor brevis (AB), adductor longus (AL), pectineus (PC). Hamstrings: biceps femoris longus (BFL), biceps femoris brevis (BFB), semimembranosus (SM), semitendinosus (ST). Gluteus: gluteus minimus (Gmi), gluteus medius (Gme), gluteus maximus (Gma). Quadriceps: rectus femoris (RF), vastus lateralis (VL), vastus medialis (VM), vastus intermedius (VI). Other: gracilis (GC), sartorius (SR), tensor fascia lata (TFL). Panel B: 3D visualisations of ground truth segmentations and model predictions for the case with the lowest DSC (56.7%), the highest DSC (96.5%) and a representative case with DSC close to the mean DSC (DSC = 92.6%).

For all images in this study, ground truth 3D segmentations for each of the 18 muscles in the upper leg (indicated in Figure 2A) were obtained by manual delineation of the OP images using ITK‐SNAP. A U‐Net [20] CNN was trained to jointly segment all muscles using the quantitative PDFF image as input, such that a single model could be trained and applied for MRI scans acquired at 1.5 T or 3 T. Five‐fold cross validation was performed for training the model and the model predictions for the validation cases for all five folds were collected to yield automated muscle segmentations for the entire dataset.

The architecture and training of the CNN was similar to the models described in Huysmans et al. [21]. The original images were split into two equal halves along the mid‐sagittal plane to separate the left leg from the right leg, and symmetry between both halves was exploited by mirroring the left leg image to mimic the right leg image, doubling the number of images available for training of the CNN. Zero‐padded patches of dimensions 216 × 216 × 81, aligned centrally along the medial/lateral and anterior/posterior axes and randomly positioned along the inferior/superior axis of the upper leg, were extracted from the images to serve as input for the network. To enhance the variability of the input data, conventional data augmentation techniques such as Gaussian noise addition and affine transformations were employed. A linear combination of cross‐entropy and Dice loss, weighted equally, was used as the loss function. The batch size was set to 4, and optimisation was performed using Adam [22] over 200 epochs with an initial learning rate of 10^−2^, which was gradually reduced over consecutive epochs [23]. Segmentation for an entire single leg image volume was obtained by combining the output of the CNN for separate, partially overlapping image patches. The output of the left leg image was subsequently mirrored and stitched to the corresponding right leg image. Per muscle only, the two largest (left and right) components of the segmentation were retained to remove small blobs of incorrectly labelled voxels and holes inside a segmented muscle volume were filled.

### 2.3 Per Muscle FF% and Mercuri Score

FF% per muscle was computed over the entire extent of each muscle by averaging the PDFF image within each entire muscle volume for the left and right leg combined, resulting in a total of 18 values per patient. FF% per muscle was also computed by only considering the muscle volume in the limited FOV as defined above. In addition, all muscles were manually graded for the left and right leg separately (36 values per patient) by a trained expert (KGC, LI) on the T1‐weighted MRI scans using the same modified Mercuri score as used by Verdú‐Díaz et al. [10]: 0 = normal appearance, 1 = mild fat replacement (< 30% of the muscle volume affected), 2 = moderate fat replacement (30%–60% of the volume), 3 = severe fat replacement (> 60% of the volume), and 4 = complete fat replacement.

### 2.4 Classification of NMDs

A random forest classifier was built to classify a symptomatic patient as either BMD, CMT1A, DM1, LGMDR12 or HC using the per muscle FF% values as features. The baseline model was trained using the 18 per muscle FF% values computed over the entire muscle extent obtained with the automated segmentation. For comparison, alternative models were trained in an identical way, but using the FF% features obtained with the manual delineation, the FF% features extracted from a limited field of view (FOV), or the 36 per muscle Mercuri scores. Patients that appeared asymptomatic on MRI were excluded from the training set. This was done by defining a threshold on the mean FF% value for each muscle group above which excess fat replacement was considered to be present for that muscle group: quadriceps: FF% ≥ 10%; hamstrings: ≥ 15%; adductors: ≥ 10%; gluteus: ≥ 20%; other: ≥ 15%. A patient was considered to be symptomatic if the average FF% in at least one of these muscle groups exceeded its respective threshold.

Furthermore, six cases with significant Dixon MRI fat‐water swap artefacts were also excluded for further analysis. The number of retained symptomatic cases used for training of the classification model is listed in Table 1 for each NMD class.

For training of the classifier, a five‐fold stratified cross‐validation approach was used to split the data set into training and validation cases. No separate test set was extracted due to the limited number of cases available. Classification performance was assessed using the one‐vs‐rest receiver operator characteristic (ROC) curve, the area under the curve (AUC) and the overall accuracy (i.e., the number of misclassified cases when assigning each case to the class with highest probability), evaluated over the entire set of validation cases for all five folds combined. The performance of different classifiers was compared using Cohen's kappa and the paired *t*‐test. Differences were considered to be significant when the associated *p*‐value was smaller than 0.05. All statistical tests were conducted using Python.

### 2.5 Interpreting Model Predictions Using SHAP Values

To better understand which muscle features are important in the discrimination of symptomatic NMD cases, SHapley Additive exPlanations (SHAP)^10^ are used. SHAP is a game theoretic approach to explain the output of any machine learning model. In essence, SHAP assigns each feature an importance value for a particular prediction, providing a rich interpretation of the model predictions in terms of feature contributions. The SHAP summary plot provides a compact and detailed summary of the overall behaviour of a machine learning model, highlighting the most important features and their range of effects on the model's predictions.

## 3 Results

### 3.1 3D Muscle Segmentation

The agreement between the automated muscle segmentations and the manual ground truth delineations was assessed by their volumetric overlap, quantified using the Dice Similarity Coefficient (DSC), yielding a mean DSC of 92.6% (range 56.7%–96.5%). Lower DSC values were in particular observed for cases with a larger amount of fat replacement. Figure 2B shows 3D visualisations of the ground truth segmentations and model predictions for the case with the lowest and the highest DSC and a case with DSC close to the mean DSC, illustrating the overall quality of the muscle segmentation.

### 3.2 Per Muscle FF% and Mercuri Score

**FIGURE 3:**
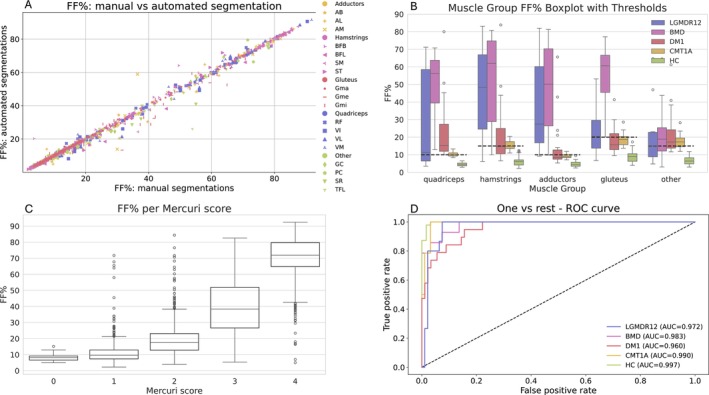
Muscle FF% quantification and classification in NMDs. Panel A: Scatter plot of per muscle FF% quantified using the manual delineation versus the automated segmentation for each case in our study (156 × 18 values). Adductors (yellow): adductor magnus (AM), adductor brevis (AB), adductor longus (AL), pectineus (PC). Hamstrings (pink): biceps femoris longus (BFL), biceps femoris brevis (BFB), semimembranosus (SM), semitendinosus (ST). Gluteus (red): gluteus minimus (Gmi), gluteus medius (Gme), gluteus maximus (Gma). Quadriceps (blue): rectus femoris (RF), vastus lateralis (VL), vastus medialis (VM), vastus intermedius (VI). Other (green): gracilis (GC), sartorius (SR), tensor fascia lata (TFL). Panel B: Boxplot of FF% values per muscle group for each NMD and HC. The FF% threshold to distinguish between symptomatic and asymptomatic cases is indicated with a black dashed line for each muscle group. Panel C: Distribution of FF% values over all muscles that were rated with the same Mercuri score. Panel D: Receiver‐operator characteristic (ROC) curves for the baseline classification model using per muscle FF% values computed using the automated muscle segmentations. Area under the curve (AUC) values for one‐vs‐rest classification are listed per class.

The FF% values computed using the automated muscle segmentation were compared with the corresponding values computed using the manual delineation, showing overall good agreement for all muscles over the entire range of FF% values (Figure 3A). Outliers were mainly due to few not well segmented cases (DSC ~ 60%). The distribution of FF% values over different muscle groups for each NMD and HC separately is shown in Figure 3B, together with the thresholds used to select the symptomatic cases used for training of the NMD classification models. The number of retained symptomatic cases for each NMD class is summarised in Table 1. Figure 3C shows the distribution of FF% values over all muscles that were rated with the same Mercuri score. Mean FF% values were 8.2%, 10.7%, 19.8%, 40.0% and 69.0% for Mercuri scores 0 to 4 respectively.

### 3.3 Classification of NMDs

**FIGURE 4:**
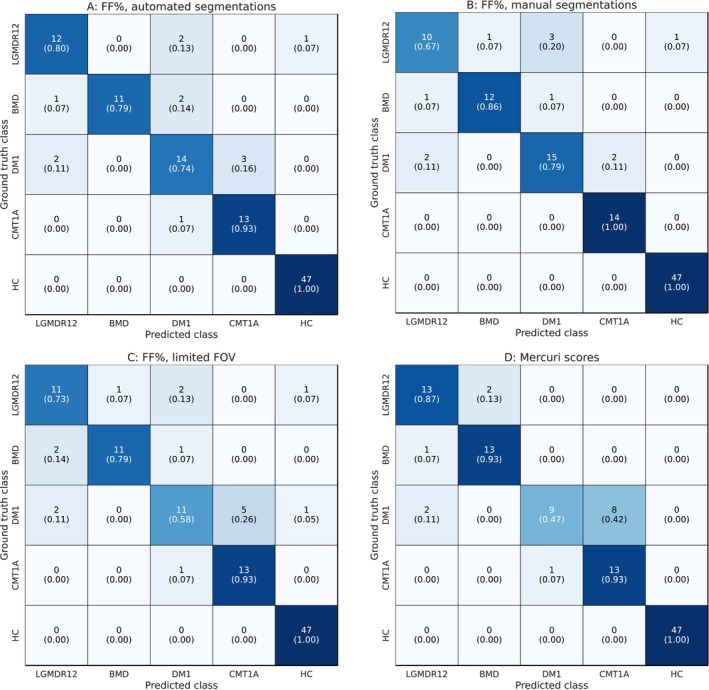
Confusion matrices for NMD classification. Confusion matrix for NMD classification using the baseline model based on per muscle FF% features computed using automated muscle segmentations (A) and identically trained alternative models using per muscle FF% computed from manual segmentations (B), a limited FOV (C) and Mercuri scores (D).

Figure 3D shows the ROC curves and AUC values for one‐versus‐rest NMD classification for the baseline model trained with per muscle FF% features computed using the automated muscle segmentation. Overall, the model demonstrates strong performance in distinguishing between different classes, as evidenced by high AUC values (≥ 0.96) for every class. The confusion matrix displayed in Figure 4A shows that 12 cases out of 109 are misclassified, resulting in an overall accuracy of 89%. The alternative models trained similarly as the baseline model but using FF% features computed from manual delineations, FF% features extracted from a limited FOV, or Mercuri scores yielded overall accuracies of 89.9% (11 misclassified cases), 85.3% (16 misclassified cases) and 87.2% (14 misclassified cases) respectively (Figure 4B–D). None of these models performed significantly differently than the baseline model (Cohen's kappa of 0.924, 0.824 and 0.886 respectively, *p* > 0.05).

### 3.4 Interpreting Model Predictions Using SHAP Values

**FIGURE 5:**
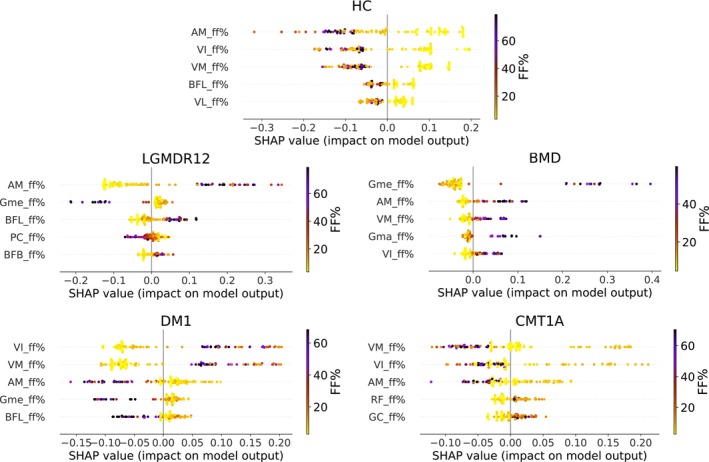
SHAP summary plot per disease class. The SHAP beeswarm summary plot illustrates the impact of the five most important features on the model's output. Each point in the plot represents a Shapley value for a feature of a subject. The features are ordered from top to bottom, with the most impactful feature listed first, by the sum of the SHAP value magnitudes over all samples, and the x location of the dot provides insight into the impact of the feature on the model output. The colour represents the value of the FF% feature from low (yellow) to high (dark purple). Muscles: adductor magnus (AM), pectineus (PC), biceps femoris longus (BFL), biceps femoris brevis (BFB), gluteus medius (Gme), gluteus maximus (Gma), rectus femoris (RF), vastus lateralis (VL), vastus medialis (VM), vastus intermedius (VI), gracilis (GC).

Figure 5 displays the SHAP summary plots for every class. When comparing the SHAP summary plots with the muscle patterns present in Table 1 it is observed that the features that are most important in explaining the behaviour of the classification model can be matched with the muscle patterns described in the medical literature. For instance, a high fat replaced adductor magnus (AM) is a positive indication for both BMD and LGMDR12, but in combination with a low fat replaced gluteus medius (Gme), the model points towards LGMDR12 and with a high fat replaced Gme, the model points towards BMD. This matches with the medical literature where glutei are generally spared in LGMDR12 but are affected from onset of the disease for BMD. For DM1, preferential involvement of the quadriceps, particularly the vastus intermedius (VI) and vastus medialis (VM), is reported in the medical literature. These two muscles also emerge as the most important features in the classification model for identifying DM1. While VI and VM are also key discriminative features in the classification of CMT1A, they are typically spared in this condition. This contrasting involvement makes VI and VM important muscles for distinguishing between DM1 and CMT1A.

## 4 Discussion

This study presents a fully automated approach that successfully differentiates between four neuromuscular disorders and healthy controls using fat‐sensitive MRI scans. The method involves automatic segmentation of 18 upper leg muscles and subsequent classification based on individual muscle fat fraction values. To improve the transparency and clinical relevance of the model, SHAP‐based interpretability techniques are applied, linking the model's decisions to known muscle involvement patterns described in medical literature.

The average segmentation performance aligns with the results reported by Huysmans et al. [21], where separate models were trained for patients with low and high levels of fat replacement. While training two models may improve segmentation quality in patients with severe fat infiltration, it introduces additional complexity to the fully automated classification pipeline, as it would require a decision mechanism to select the appropriate segmentation model per patient. In contrast, our approach uses a single segmentation model for all patients simplifying the classification workflow without significantly compromising performance.

The classification accuracy achieved in our study is consistent with previously reported results in the literature; Verdú‐Diaz et al. [10] reported a high accuracy of 95.7% using a random forest classifier trained on Mercuri scores from 976 lower limb MRIs, covering 70 muscles, and distinguishing between 10 different NMDs. While their model benefited from a substantially larger dataset and the inclusion of more muscles compared with our study, it relied on expert derived Mercuri scores, introducing subjectivity and limiting scalability. In contrast, our baseline model uses more objective and automated features and achieved an accuracy of 89% in a five‐class classification problem. Other deep learning–based studies, such as Cai et al. [11] and Yang et al. [13], reported accuracies of 90.7% and 91%, respectively, but both focused on binary classification tasks. Similarly, Rodrigues et al. [24] achieved 93.8% accuracy in a three‐class classification problem.

In terms of model explainability, our study leverages SHAP values to provide detailed, per‐class insights into feature importance, allowing for direct interpretation of how specific muscle involvement contributes to classification outcomes. In addition to global interpretability, SHAP values can also provide per‐case insights, offering transparency into individual predictions. This contrasts with the Gini index used by Verdú‐Diaz et al. [10], which offers a more global and less interpretable measure of feature relevance. Cai et al. [11] and Yang et al. [13] used class activation mapping (CAM) to visualise discriminative regions in MRI images, while Rodrigues et al. [24] did not report any explainability analysis. The use of SHAP in our study thus adds to the transparency of our classification model, aligning model decisions with known disease patterns and enhancing clinical interpretability.

While it may seem intuitive that muscles identified in Table 2 as characteristically affected or spared should consistently rank highest in the SHAP summary plots, this is not necessary the case in a multi‐class classification setting. The SHAP values reflect the contribution of each feature (FF%) to the model's decision‐making process for distinguishing between different NMDs. Therefore, muscles that are uniformly affected or spared across multiple diseases may have limited discriminative power and thus receive lower SHAP importance scores. In contrast, muscles that exhibit contrasting involvement patters between specific disease pairs, such as gluteus medius being spared in LGMDR12 but affected in BMD, are more informative for classification and consequently rank higher in SHAP importance. This explains why some muscles not highlighted in Table 2 may appear more prominently in the SHAP plots, while others that are consistently involved across disease may appear less frequently or with lower importance.

The interpretability of the features used by the classification model allows us to investigate the misclassified cases in the confusion matrix shown in Figure 4A. Two of the three misclassified BMD cases are the two cases with the overall lowest automated segmentation quality (DSC of 56.7% and 61.9% respectively), resulting in a less reliable quantification of the per muscle fat content in these cases. For all other misclassified cases, the DSC of the automated muscle segmentation is above 90%. The one LGMDR12 case that is misclassified as HC is a case where only the adductor magnus shows slight fat replacement (FF% ~ 20%), which is somewhat underestimated when using the automated segmentation (FF% ~ 17%), which leads to a misclassification as HC. The model misclassifies three DM1 cases as CMT1A and one CMT1A case as DM1. As can be seen in the SHAP summary plot for the DM1 and CMT1A cases, the vastus muscles are the most significant in explaining the model's decision. The distinction between CMT1A and DM1 is primarily due to the degree of fat replacement in the vastus medialis (VM) and the vastus intermedius (VI): Lower FF% values suggest a higher likelihood for CMT1A, while higher values indicate a greater probability for DM1. For the three misclassified DM1 cases, a lesser degree of fat infiltration in the vastus muscles is present (< 15%), which ultimately leads to a misclassification as CMT1A. The model also misclassified two LGMDR12 cases as DM1 cases and two DM1 cases as LGMDR12 cases. These two misclassified DM1 cases are the two cases with the highest overall fat replacement present in the DM1 dataset, while the two misclassified LGMDR12 cases are two early‐stage cases with relatively low fat replacement. This indicates that the model has difficulty to differentiate an end‐stage DM1 patient from a LGMDR12 patient and an early‐stage LGMDR12 patient from a DM1 patient.

The baseline model demonstrates comparable performance to the Mercuri score model in terms of overall statistics. However, analysis of the confusion matrices reveals that the Mercuri score model struggles more with distinguishing between CMT1A and DM1, misclassifying eight DM1 cases as CMT1A, compared with three misclassifications by the baseline model. SHAP summary plots identify the vastus intermedius (VI) and vastus medialis (VM) as the most critical muscles influencing the model's decisions. For the CMT1A cases correctly classified by the Mercuri score model, both VM and VI consistently have a Mercuri score of 1, whereas they never have a score of 1 for correctly classified DM1 cases. In contrast, all misclassified DM1 cases (except one, where the left VM muscle has a score of 2) have a Mercuri score of 1 for both VM and VI. The baseline model misclassifies only three of the eight cases that the Mercuri score model misclassifies, suggesting that FF% (ranging from 0 to 100%) is more effective in capturing subtle differences between DM1 and CMT1A compared with the categorical Mercuri score.

We demonstrated that satisfactory MRI‐based NMD classification performance can still be achieved using FF% values computed from a limited FOV. This finding supports the application of this model in the clinical environment where time constraints may prevent the acquisition of three Dixon stacks to cover the entire upper leg. Even though Cohen's kappa and the paired *t*‐test show that the performance of the models trained on the limited FOV is not statistically different from the baseline model using the full FOV, we do note a small drop in accuracy (16 misclassified cases vs. 12). Model performance can be improved by including additional metrics such as per muscle volume%, that is, the volume of a specific muscle divided by the total volume of muscle present in the scan. When adding these features to the limited FOV model, the accuracy was found to increase from 85.3% to 89%. Furthermore, certain neuromuscular disorders do not exhibit uniform progression, implying that the rate of fat replacement is not identical in the left/right or proximal/distal sections of a muscle. The FF% values as used in this study do not account for this asymmetry. Features such as for instance the absolute difference in FF% between the left and right muscle to quantify lateral asymmetry, or the absolute difference in FF% between the proximal and distal halves of a muscle to account for longitudinal asymmetry can provide additional information to the classification models. The choice to only incorporate global per muscle FF% features in the baseline model was made intentionally for the current study to facilitate straightforward interpretation using SHAP explanations.

The decision to focus on the proximal legs in this study was primarily driven by the inclusion of LGMDR12 and BMD patients in our initial natural history studies, in whom fat replacement is known to predominantly affect proximal muscles. Hence, these studies did not include distal leg images for LGMDR12 patients, while for BMD patients, only the right leg was imaged. Our studies involving CMT1A and DM1, which typically affect more distal muscle groups, were initiated later, and included both proximal and distal leg images. Our analysis was thus restricted to the proximal legs, as distal leg images were not available for all cases. Extending the model to include additional per muscle features can be investigated in future work.

The main limitation of the current study is the relatively small dataset size, which included 47 HC cases and 62 cases from four different NMDs compared with 986 cases from 10 NMDs used in Verdú‐Díaz et al. [10]). Due to the small number of cases available per NMD, it was not feasible to extract a separate test set. If more data would be available, it would be worthwhile to employ a separate test set and explore whether a larger training dataset would lead to an improvement in classification performance.

The classification model developed in this study has the potential to support clinical decision‐making by providing rapid, quantitative insights into disease patterns based on MRI‐derived fat fraction data. Once MRI scans are acquired, the model can generate diagnostic suggestions almost instantly, offering a valuable complement to slower diagnostic tools such as genetic testing. This capability could help prioritise further investigation, guide early therapeutic decisions and reduce reliance on invasive procedures like muscle biopsy. However, to effectively utilise classification models in clinical practice, a broader range of NMDs must be represented. This remains challenging due to the rarity of many NMDs and the need for patients to be sufficiently progressed in their disease for inclusion. Additionally, while Dixon MRI is essential for accurate fat fraction quantification, it is less commonly used in routine clinical practice compared with standard T1‐weighted imaging, posing another barrier to widespread adoption.

## 5 Conclusion

The findings of this study demonstrate that a fully automated method, which involves automatic segmentation of 18 muscles in the upper leg in fat‐sensitive MRI scans followed by classification of each image using per muscle fat fraction percentages, is effective in distinguishing between four NMDs and HC. To enhance the interpretability of the classification model, SHAP explanations are provided, which can be associated with muscle patterns documented in the medical literature for the NMDs considered in this study. This classification model therefore has the potential to serve as a clinically relevant tool to support MRI‐based diagnosis of NMDs.

## Disclosure

K.G.C. is Chairholder of the Emil von Behring Chair for Neuromuscular and Neurodegenerative Disorders by CSL Behring. K.G.C. is member of the European Reference Network for Rare Neuromuscular Diseases (ERN EURO‐NMD) and of the European Reference Network for Rare Neurological Diseases (ERN‐RND). The authors report no disclosures relevant to the manuscript.

## Conflicts of Interest

The authors declare no conflicts of interest.

## Data Availability

The anonymised supporting datasets analysed during the current study can be made available to qualified investigators from the corresponding author on reasonable request.
